# Analysis of risk factors associated with the development and postoperative complications of complicated acute appendicitis in elderly patients

**DOI:** 10.3389/fsurg.2025.1673385

**Published:** 2025-12-03

**Authors:** Zhesi Jin, Qian Zhang, Huazhong Cai

**Affiliations:** Department of Emergency, Affiliated Hospital of Jiangsu University, Zhenjiang, Jiangsu, China

**Keywords:** acute appendicitis, complicated, elderly, postoperative complications, risk factors

## Abstract

**Objective:**

Based on an analysis of large-scale retrospective case data, this study aimed to identify the risk factors associated with the development and postoperative complications of complicated acute appendicitis (CAA) in elderly patients (>60 years).

**Methods:**

A total of 296 elderly patients diagnosed with acute appendicitis (AA) who underwent appendectomies at our hospital between January 2020 and January 2025 were enrolled in this study. These patients were categorized into either the CAA group (*n* = 113) or the uncomplicated acute appendicitis (UCAA) group (*n* = 183), based on the severity of their clinical presentation. Subsequently, univariate and multivariate logistic regression analyses were performed to identify the risk factors associated with the onset of CAA and its postoperative complications.

**Results:**

The elderly patients in the CAA group exhibited a higher risk of postoperative complications and intensive care unit (ICU) admission, as well as prolonged hospitalization, compared to those in the UCAA group. Preoperative abdominal pain lasting more than 3 days [odds ratio (OR) = 3.159, *P* = 0.038], the presence of abdominal muscle tension (OR = 2.297, *P* = 0.007), appendiceal fecalith (OR = 2.697, *P* = 0.002), temperature ≥ 37.45 °C (OR = 2.968, *P* = 0.001), neutrophil percentage ≥ 82.7% (OR = 2.593, *P* = 0.010), and C-reactive protein (CRP) level ≥ 4.3 mg/L (OR = 3.256, *P* < 0.001) were identified as independent risk factors associated with the development of CAA. The incidence of postoperative complications in the elderly patients in the CAA group was 31%, which was significantly higher than the 6.0% observed in the UCAA group. An analysis based on the data from the patients with CAA indicated that the presence of nausea/vomiting (OR = 3.629, *P* = 0.033), white WBC ≥ 14.24 × 10^9^/L (OR = 3.825, *P* = 0.021), neutrophil percentage ≥ 84.3% (OR = 11.165, *P* = 0.012), and open appendectomy (OR = 5.799, *P* = 0.002) were independent risk factors for postoperative complications.

**Conclusions:**

Abdominal signs and symptoms, the presence of appendicoliths, body temperature, and the levels of neutrophils and CRP were associated with the occurrence of CAA, while surgical approaches and the levels of WBCs and neutrophils were associated with postoperative complications. This study explored the risk factors associated with CAA and its postoperative complications in elderly patients, thereby offering valuable insights for the clinical management and treatment of AA in this population.

## 1 Introduction

Acute appendicitis (AA) is one of the most prevalent acute abdominal diseases and represents the leading cause of emergency abdominal surgical intervention. AA can be classified into the following five subtypes based on clinical features and pathological changes: simple, suppurative, gangrenous, perforated appendicitis, and periappendiceal abscess (1, 2). The first two subtypes are categorized as uncomplicated acute appendicitis (UCAA), whereas the latter three are collectively referred to as complicated acute appendicitis (CAA). CAA accounts for approximately 20%–30% of all AA cases. Because CAA is refractory to conservative treatment, it is also referred to as irreversible appendicitis (3, 4).

Since the 19th century, an appendectomy has been the established standard treatment for AA. However, recent clinical trials have demonstrated that antibiotic therapy constitutes a viable and effective alternative for cases of UCAA (5). The hospitalization rate for UCAA has declined due to the impact of the novel coronavirus pneumonia (COVID-19), and the negative appendectomy rate has also significantly decreased (6, 7). These changes suggest that conservative treatment for UCAA may help reduce the incidence of negative appendectomies, shorten hospital stays, and lower medical costs. Patients with CAA require different management compared to those with UCAA; however, differentiating between the two forms remains a clinically challenging task (8).

AA in elderly patients represents a distinct clinical entity. Due to the diminished physiological response capacity associated with aging, the severity of the pathological changes often does not correspond with the presenting symptoms and physical signs. This discrepancy can pose significant challenges in both diagnosis and management (9, 10). Delayed intervention for CAA in elderly patients may result in severe complications, including potentially life-threatening outcomes. Currently, there remains a paucity of research on risk factor analysis and intervention strategies related to the onset and postoperative complications of CAA among the elderly population (10, 11). This study investigated the risk factors associated with the development and postoperative complications of CAA in elderly patients, based on clinical symptoms, signs, and results from laboratory and imaging examinations, with the aim of providing a reference to reduce the misdiagnosis and missed diagnosis of this condition.

## 2 Materials and methods

### 2.1 Study participants

This retrospective observational study included elderly patients with AA who underwent appendectomies at our hospital between January 2020 and January 2025. Relevant clinical data were collected through the electronic medical record system. Subsequently, the research participants were selected based on the predefined inclusion and exclusion criteria. The inclusion criteria were as follows: (1) age >60 years (9); (2) patients who underwent a laparoscopic or open appendectomy; (3) patient's first onset of illness; and (4) pathological type was confirmed through both intraoperative and postoperative histopathological analyses. The exclusion criteria were as follows: (1) patients ≤60 years old and pregnant women; (2) patients with concurrent cardiopulmonary insufficiency, malignant neoplasms, or disorders of the immune and hematological systems; (3) patients whose postoperative pathological findings indicated the presence of appendiceal tumors; and (4) patients with incomplete medical records, examination results, and pathological findings. This study was reviewed and approved by the ethics committee of the Affiliated Hospital of Jiangsu University and was performed in accordance with the Declaration of Helsinki.

### 2.2 Data collection

The dependent variables examined in this study included the incidence of CAA and postoperative complications. Moreover, the demographic and clinical characteristics that were collected and analyzed in this study include gender, age, body mass index (BMI), history of smoking and alcohol consumption, preoperative antibiotic therapy, preoperative abdominal pain duration, presence of diarrhea, nausea/vomiting, the migration of the abdominal pain from the upper to the right lower abdomen, abdominal muscle tension, postoperative complications, transferred to intensive care unit (ICU), surgical approach, and hospital stay. The analysis included the assessments of body temperature, serum white blood cell (WBC) count, platelet (PLT) count, neutrophil percentage, C-reactive protein (CRP), and total and direct bilirubin levels. Abdominal muscle tension is a clinical sign resulting from irritation of the parietal peritoneum due to inflammatory processes. The indicators requiring evaluation using ultrasound and computed tomography (CT) imaging examinations included the appendix diameter, presence of extraneous appendiceal free gas, fluid accumulation around the appendix, and appendiceal fecalith. The appendix diameter was measured in both the longitudinal and transverse sections of the image. On the longitudinal section of the appendix, the distance perpendicular to the serosal layer from one side to the opposing serosal layer was measured. On the transverse section, the outer diameter of the concentric circular structure was measured. An open appendectomy refers to a surgical procedure in which patients are operated on primarily using the open technique.

### 2.3 Outcome evaluation

The types of AA were diagnosed based on intraoperative and postoperative pathological findings, which are regarded as the gold standard. UCAA is defined as a condition in which the appendix appears red, swollen, and thickened during surgical exploration, and a postoperative pathological examination confirms the diagnosis of simple or suppurative appendicitis. CAA is defined as a condition in which the appendix appears blackened, necrotic, or perforated during surgical exploration, and is subsequently confirmed by a postoperative pathological examination to be gangrenous or perforated appendicitis (3, 8). All the postoperative complications identified in this study occurred within the 30-day period following the appendectomy and included fever, infection, gastrointestinal dysfunction, and intestinal fistula. The Clavien-Dindo classification system (2009 version), one of the most widely recognized international standards, was employed to evaluate the postoperative complications (12).

### 2.4 Statistical analysis

All the statistical analyses in this study were conducted using SPSS software (version 26.0). The normal distribution of continuous data was assessed by constructing a histogram. If the data conformed to a normal distribution, the mean ± standard deviation was computed, and an independent-samples T-test was used for inter-group comparisons. Otherwise, the median (interquartile range) was reported, and the Mann–Whitney *U*-test was employed. Categorical data were presented as frequencies (percentages) and inter-group differences were analyzed using the two-tailed chi-square test. Additionally, receiver operating characteristic (ROC) curves were generated for body temperature, serum WBC and PLT counts, neutrophil percentage, CRP, total bilirubin, direct bilirubin level, and appendiceal diameter. The cut-off value was defined as the threshold corresponding to the maximum sum of the sensitivity and specificity values, and was applied in the subsequent conversion of continuous variables. Univariate and multivariate binary logistic regression analyses were performed to identify the predictive factors associated with the incidence of CAA and postoperative complications among the elderly population. The sample size in this study was limited. To minimize the risk of overlooking potentially significant predictive variables (Type II error) during the development of the multivariate model, variables with a *P*-value < 0.1 in the univariate analysis were selected for inclusion in the multivariate regression model.

## 3 Results

### 3.1 Patient characteristics

**Figure 1:**
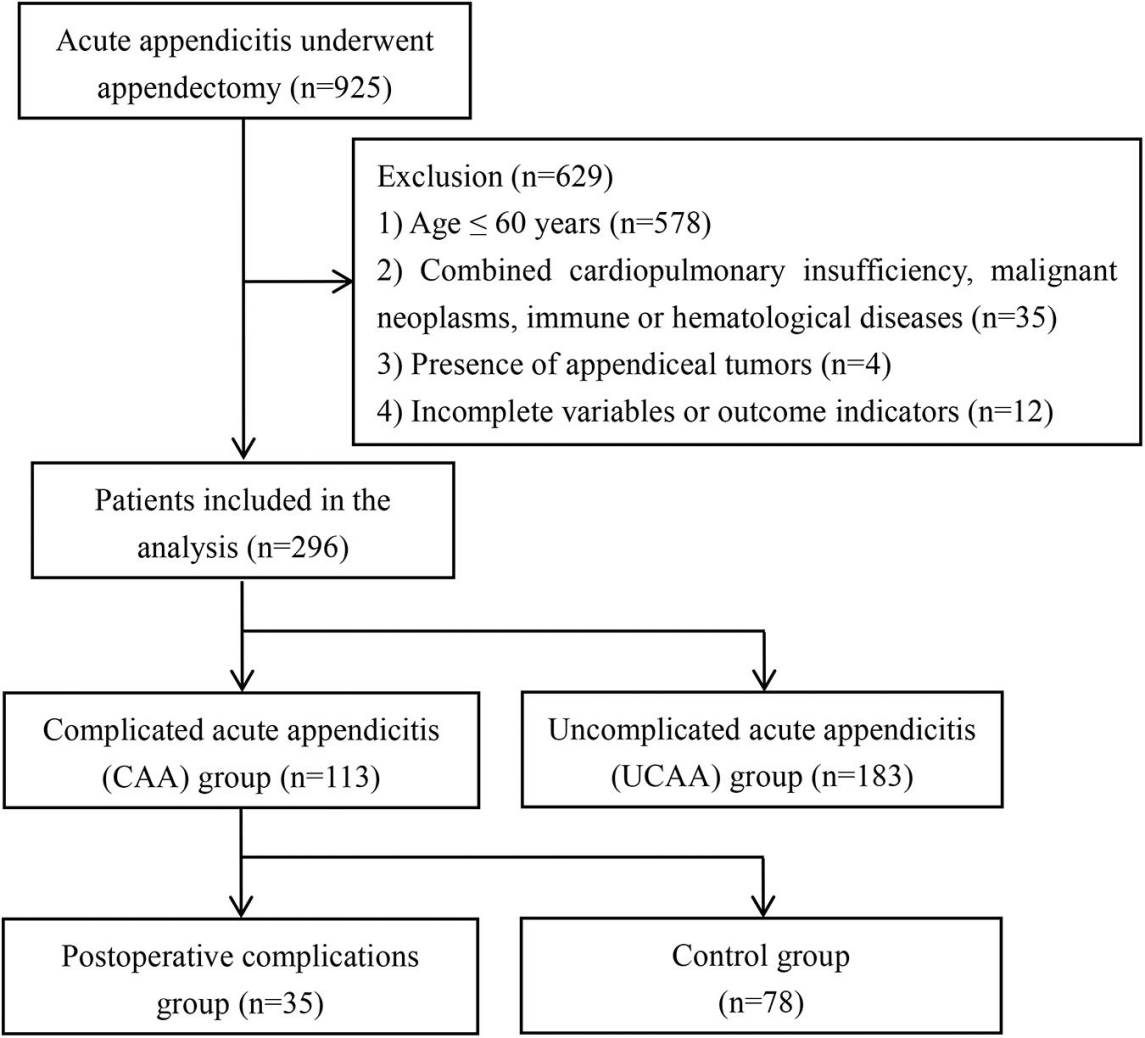
The flowchart of the patient screening process.

A total of 925 patients with AA underwent appendectomies at our hospital between 2020 and 2025. After rigorous screening, 296 elderly AA patients were included in this retrospective case-control study. These patients were categorized into either the CAA group (*n* = 113) or the UCAA group (*n* = 183), based on their intraoperative and postoperative pathological findings (Figure 1). The incidence rate of CAA in this elderly population reached 38.2%. The elderly patients in the CAA group exhibited a higher risk of postoperative complications (*P* < 0.001) and ICU admission (*P* < 0.001), as well as prolonged hospitalization (*P* < 0.001), compared to those in the UCAA group (Table 1). The post-appendectomy complications identified in this study included fever, surgical site infection, pulmonary infection, intra-abdominal infection, gastrointestinal dysfunction, and intestinal fistula.

**Table 1:** Comparison of the demographic and clinical characteristics of elderly patients with complicated or uncomplicated acute appendicitis (univariate analysis).

Characteristic	Total (*n* = 296)	CAA group (*n* = 113)	UCAA group (*n* = 183)	*P*-value
Gender				0.217
Male	170 (57.4)	70 (61.9)	100 (54.6)	
Female	126 (42.6)	43 (38.1)	83 (45.4)	
BMI (kg/m^2^)	24.01 ± 3.16	23.81 ± 3.06	24.14 ± 3.22	0.373
Diabetes				0.257
Presence	34 (11.5)	16 (14.2)	18 (9.8)	
Absence	262 (88.5)	97 (85.8)	165 (90.2)	
Smoking history				0.328
Presence	67 (22.6)	29 (25.7)	38 (20.8)	
Absence	229 (77.4)	84 (74.3)	145 (79.2)	
Drinking history				0.820
Presence	94 (31.8)	35 (31.0)	59 (32.2)	
Absence	202 (68.2)	78 (69.0)	124 (67.8)	
Preoperative antibiotic therapy				0.486
Presence	243 (82.1)	95 (84.1)	148 (80.9)	
Absence	53 (17.9)	18 (15.9)	35 (19.1)	
Abdominal pain before operation (days)				**<0**.**001**
≤1	64 (21.6)	12 (10.6)	52 (28.4)	
>1, ≤3	182 (61.5)	72 (63.7)	110 (60.1)	
>3	50 (16.9)	29 (25.7)	21 (11.5)	
Diarrhea				**0**.**005**
Presence	27 (9.1)	17 (15.0)	10 (5.5)	
Absence	269 (90.9)	96 (85.0)	173 (94.5)	
Nausea/vomiting				0.080
Presence	167 (56.4)	71 (62.8)	96 (52.5)	
Absence	129 (43.6)	42 (37.2)	87 (47.5)	
Migrating abdominal pain				0.226
Presence	231 (78.0)	84 (74.3)	147 (80.3)	
Absence	65 (22.0)	29 (25.7)	36 (19.7)	
Abdominal muscle tension				**<0**.**001**
Presence	151 (51.0)	80 (70.8)	71 (38.8)	
Absence	145 (49.0)	33 (29.2)	112 (61.2)	
Temperature (°C)	37.57 ± 1.12	37.95 ± 1.16	37.34 ± 1.02	**<0**.**001**
WBC (10^9^/L)	13.31 ± 4.52	14.14 ± 4.85	12.79 ± 4.24	**0**.**012**
Neutrophil (%)	84.68 ± 7.91	86.82 ± 6.22	83.35 ± 8.55	**<0**.**001**
CRP (mg/L)	5.61 (0.77, 6.99)	5.61 (5.39, 10.91)	3.29 (0.20, 5.61)	**<0**.**001**
PLT (10^9^/L)		197.27 ± 65.81	194.31 ± 47.48	0.678
Total bilirubin (μmol/L)	17.00 (12.25, 23.78)	18.70 (14.55, 28.05)	15.60 (10.80, 21.90)	**0**.**001**
Direct bilirubin (μmol/L)	5.40 (3.60, 8.00)	6.40 (4.15, 10.40)	5.10 (3.30, 6.80)	**<0**.**001**
Extraneous appendiceal free gas				**0**.**008**
Presence	13 (4.4)	10 (8.8)	3 (1.6)	
Absence	283 (95.6)	103 (91.2)	180 (98.4)	
Fluid accumulation around the appendix				**<0**.**001**
Presence	79 (26.7)	45 (39.8)	34 (18.6)	
Absence	217 (73.3)	68 (60.2)	149 (81.4)	
Appendiceal fecalith				**<0**.**001**
Presence	149 (50.3)	76 (67.3)	73 (39.9)	
Absence	147 (49.7)	37 (32.7)	110 (60.1)	
Appendix diameter (mm)	12.79 ± 3.99	14.13 ± 4.91	11.97 ± 3.03	**<0**.**001**
Postoperative complications				**<0**.**001**
Presence	46 (15.5)	35 (31.0)	11 (6.0)	
Absence	250 (84.5)	78 (69.0)	172 (94.0)	
Transferred to the ICU				**<0**.**001**
Presence	34 (11.5)	23 (20.4)	11 (6.0)	
Absence	262 (88.5)	90 (79.6)	172 (94.0)	
Hospital stay (days)	4 (3, 7)	7 (4, 9)	4 (2, 5)	**<0**.**001**

Values in bold denote statistical significance at *P* ≤ 0.05.

### 3.2 Risk factors associated with CAA in the elderly patients

**Figure 2:**
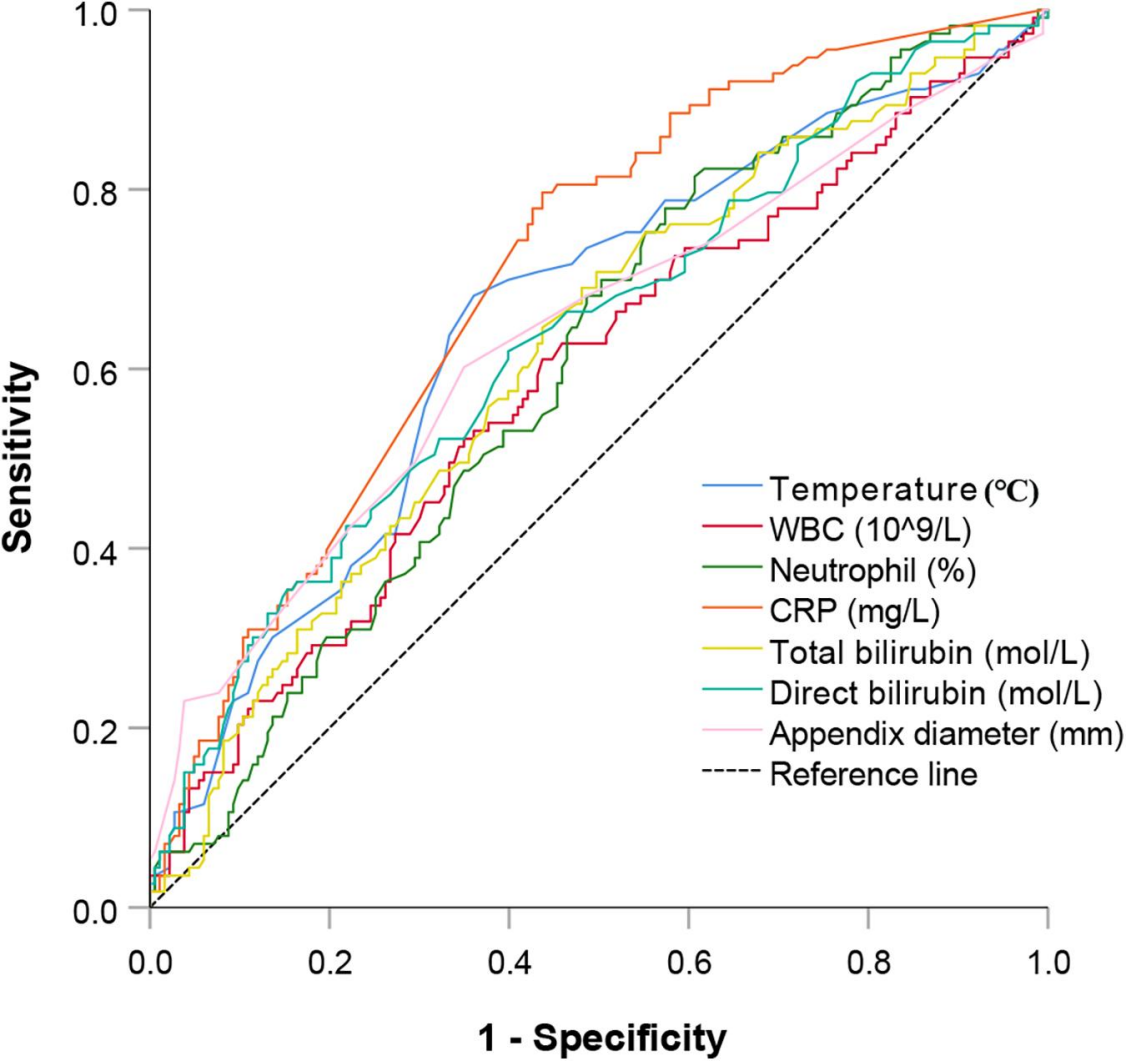
ROC curves for body temperature, serum WBC count, neutrophil percentage, CRP level, total bilirubin, direct bilirubin, and appendiceal diameter were constructed to evaluate their predictive value for the occurrence of CAA in elderly patients with AA. ROC, receiver operating characteristic; WBC, white blood cell; CRP, C-reactive protein; CAA, complicated acute appendicitis; AA, acute appendicitis.

We conducted both univariate and multivariate logistic regression analyses to evaluate the risk factors associated with CAA in a cohort of 296 elderly patients diagnosed with AA. The results of the univariate analysis indicated that the onset of CAA was significantly correlated with several preoperative clinical, laboratory, and imaging examination indicators, including duration of preoperative abdominal pain (*P* < 0.001), presence of diarrhea (*P* = 0.005), abdominal muscle tension (*P* < 0.001), body temperature (*P* < 0.001), serum WBC count (*P* = 0.012), neutrophil percentage (*P* < 0.001), CRP (*P* < 0.001), total (*P* = 0.001) and direct bilirubin levels (*P* < 0.001), appendiceal diameter (*P* < 0.001), presence of extraneous appendiceal free gas (*P* = 0.008), periappendiceal effusion (*P* < 0.001), and appendiceal fecalith (*P* < 0.001). The cut-off values for body temperature, serum WBC count, neutrophil percentage, CRP, total bilirubin level, direct bilirubin level, and appendiceal diameter were determined by plotting the ROC curves, followed by conversion of the above outcome indicators into binary variables (Figure 2). Subsequently, the transformed binary variables were incorporated into the multivariate logistic regression model. Preoperative abdominal pain lasting more than 3 days [odds ratio (OR) = 3.159, *P* = 0.038], the presence of abdominal muscle tension (OR = 2.297, *P* = 0.007), appendiceal fecalith (OR = 2.697, *P* = 0.002), temperature ≥ 37.45 °C (OR = 2.968, *P* = 0.001), neutrophil percentage ≥ 82.7% (OR = 2.593, *P* = 0.010), and CRP level ≥ 4.3 mg/L (OR = 3.256, *P* < 0.001) were identified as independent risk factors associated with the development of CAA (Table 2).

**Table 2:** Multivariate logistic regression analysis of risk factors associated with complicated acute appendicitis in elderly patients.

Variable	OR (95% CI)	*P-*value
Abdominal pain before operation (days)		0.111
≤1	Reference	
>1, ≤3	1.544 (0.673, 3.542)	0.305
>3	3.159 (1.063, 9.386)	**0**.**038**
Diarrhea		0.242
Absence	Reference	
Presence	1.925 (0.643, 5.762)	
Nausea/vomiting		0.342
Absence	Reference	
Presence	1.344 (0.730, 2.474)	
Abdominal muscle tension		**0**.**007**
Absence	Reference	
Presence	2.297 (1.253, 4.208)	
Temperature (°C)		**0**.**001**
<37.45	Reference	
≥37.45	2.968 (1.605, 5.488)	
WBC (10^9^/L)		0.198
<13.39	Reference	
≥13.39	1.490 (0.812, 2.734)	
Neutrophil (%)		**0**.**010**
<82.7	Reference	
≥82.7	2.593 (1.258, 5.345)	
CRP (mg/L)		**<0**.**001**
<4.3	Reference	
≥4.3	3.256 (1.677, 6.321)	
Total bilirubin (μmol/L)		0.486
<15.7	Reference	
≥15.7	1.359 (0.574, 3.216)	
Direct bilirubin (μmol/L)		0.850
<5.55	Reference	
≥5.55	1.084 (0.469, 2.509)	
Extraneous appendiceal free gas		0.824
Absence	Reference	
Presence	1.188 (0.260, 5.420)	
Fluid accumulation around the appendix		0.263
Absence	Reference	
Presence	1.475 (0.747, 2.912)	
Appendiceal fecalith		**0**.**002**
Absence	Reference	
Presence	2.697 (1.449, 5.019)	
Appendix diameter (mm)		0.217
<12.5	Reference	
≥12.5	1.481 (0.794, 2.761)	

Values in bold denote statistical significance at *P* ≤ 0.05.

### 3.3 Risk factors for postoperative complications in elderly patients with CAA

The incidence of postoperative complications in the CAA group was 31% (35/113), which was significantly higher than the 6.0% (11/183) observed in the UCAA group. The ROC curves for body temperature, serum WBC and PLT count, neutrophil percentage, appendiceal diameter, CRP, total and direct bilirubin levels were constructed using the incidence of postoperative complications as the dependent variable. The aforementioned continuous variables were dichotomized based on their respective cut-off values. Following the initial univariate analysis, gender, presence of nausea/vomiting, abdominal muscle tension, body temperature ≥ 38.95 °C, WBC ≥ 14.24 × 10^9^/L, neutrophil percentage ≥ 84.3%, CRP ≥ 4.64 mg/L, PLT ≤ 150 × 10^9^/L, direct bilirubin ≥ 10.35*μ*mol/L, appendix diameter ≥ 14.5 mm, and surgical approach were selected for inclusion in the multivariable regression model. The analysis of the data from the patients with CAA indicated that the presence of nausea/vomiting (OR = 3.629, *P* = 0.033), WBC ≥ 14.24 × 10^9^/L (OR = 3.825, *P* = 0.021), neutrophil percentage ≥ 84.3% (OR = 11.165, *P* = 0.012), and open appendectomy (OR = 5.799, *P* = 0.002) were independent risk factors for postoperative complications (Table 3).

**Table 3:** Univariate and multivariate logistic regression analyses of risk factors associated with postoperative complications in elderly patients with complicated acute appendicitis.

Variable	Univariate analysis		Multivariate analysis	
	OR (95% CI)	*P*-value	OR (95% CI)	*P*-value
Gender		0.074		0.348
Male	Reference		Reference	
Female	0.448 (0.186, 1.080)		0.562 (0.169, 1.870)	
BMI (kg/m^2^)		0.983		
≥18.5, ≤28	Reference			
<18.5	1.113 (0.193, 6.401)	0.905		
>28	0.890 (0.164, 4.843)	0.893		
Diabetes		0.238		
Absence	Reference			
Presence	1.917 (0.650, 5.649)			
Smoking history		0.349		
Absence	Reference			
Presence	1.528 (0.629, 3.709)			
Drinking history		0.167		
Absence	Reference			
Presence	1.810 (0.780, 4.197)			
Preoperative antibiotic therapy		0.430		
Absence	Reference			
Presence	0.657 (0.231, 1.868)			
Abdominal pain before operation (days)		0.269		
≤1	Reference			
>1, ≤3	2.059 (0.415, 10.207)	0.377		
>3	3.529 (0.652, 19.099)	0.143		
Diarrhea		0.327		
Absence	Reference			
Presence	1.700 (0.588, 4.914)			
Nausea/vomiting		**0**.**014**		**0**.**033**
Absence	Reference		Reference	
Presence	3.256 (1.271, 8.341)		3.629 (1.111, 11.854)	
Abdominal muscle tension		0.064		0.068
Absence	Reference		Reference	
Presence	2.559 (0.946, 6.922)		3.380 (0.912, 12.525)	
Temperature (°C)		**0**.**005**		0.084
<38.95	Reference		Reference	
≥38.95	3.667 (1.470, 9.147)		2.902 (0.868, 9.701)	
WBC (10^9^/L)		**0**.**043**		**0**.**021**
<14.24	Reference		Reference	
≥14.24	2.355 (1.028, 5.392)		3.825 (1.224, 11.957)	
Neutrophil (%)		**0**.**005**		**0**.**012**
<84.3	Reference		Reference	
≥84.3	8.735 (1.946, 39.211)		11.165 (1.717, 72.588)	
CRP (mg/L)		0.054		0.438
<4.64	Reference		Reference	
≥4.64	2.830 (0.981, 8.165)		1.841 (0.394, 8.601)	
PLT (10^9^/L)		0.060		0.375
>150	Reference		Reference	
≤150	2.385 (0.964, 5.904)		1.873 (0.469, 7.487)	
Total bilirubin (μmol/L)		0.196		
<33.9	Reference			
≥33.9	1.904 (0.717, 5.053)			
Direct bilirubin (μmol/L)		0.065		0.538
<10.35	Reference		Reference	
≥10.35	2.290 (0.951, 5.513)		1.513 (0.405, 5.647)	
Extraneous appendiceal free gas		0.944		
Absence	Reference			
Presence	0.951 (0.231, 3.916)			
Fluid accumulation around the appendix		0.205		
Absence	Reference			
Presence	1.687 (0.752, 3.785)			
Appendiceal fecalith		0.527		
Absence	Reference			
Presence	1.324 (0.555, 3.156)			
Appendix diameter (mm)		**0**.**037**		0.816
<14.5	Reference		Reference	
≥14.5	2.381 (1.055, 5.372)		1.138 (0.383, 3.375)	
Surgical approach		**0**.**022**		**0**.**002**
Laparoscopy	Reference		Reference	
Open	2.681 (1.155, 6.219)		5.799 (1.890, 17.793)	

Values in bold denote statistical significance at *P* ≤ 0.05.

## 4 Discussion

In recent years, as the elderly population has increased, AA has emerged as one of the most prevalent surgical conditions among the elderly population. Due to their slower reaction times, reduced sensitivity to pain stimuli, weakened abdominal musculature, and diminished defensive responses, elderly patients with AA often present with vague symptoms, atypical physical signs, and a lack of correlation between clinical manifestations and underlying pathological changes (10). The presence of atypical clinical manifestations and multiple comorbidities, and the concerns expressed by patients, their families, and even physicians regarding surgical intervention, contribute to the relatively delayed treatment of elderly patients with AA. Therefore, elderly patients with AA are at higher risk of developing appendiceal gangrene and perforation, and experience a greater incidence of postoperative complications, which can exacerbate their clinical condition and result in an unfavorable prognosis (13, 14). This study aimed to identify risk factors associated with the development of CAA and postoperative complications among elderly patients (>60 years) by conducting a retrospective analysis of large-scale clinical data. The objective was to enable the early identification of high-risk patients who are suitable for surgical intervention, thereby improving surgical outcomes while maintaining safety.

Preoperative abdominal pain lasting more than 3 days, the presence of abdominal muscle tension, and appendiceal fecalith were proven to be independently associated with CAA. The etiological theory of AA posits that the condition is initiated by a luminal obstruction, which results in elevated intraluminal pressure. This pressure subsequently causes mucosal injury and facilitates bacterial invasion. As the inflammatory process progresses, both the blood supply and lymphatic drainage of the appendix become compromised, ultimately leading to gangrenous changes and potential perforation (15, 16). Appendiceal calculi and benign and malignant tumors are the primary causes of appendiceal lumen obstruction in elderly individuals. Similarly, fecalith impaction can lead to a rapid increase in intraluminal pressure within the appendix, thereby exacerbating appendiceal ischemia and promoting the progression of AA (17, 18). Previous studies have also indicated that the duration of symptoms at the time of presentation constitutes an independent risk factor for the development of appendiceal gangrene or perforation (16, 19). A survey of patients with appendicitis revealed that since the COVID-19 pandemic in 2020, there has been a significant decrease in their willingness to seek medical care (*P* < 0.001). The time interval from symptom onset to hospital admission increased to 65.0 h, which was significantly longer compared to the pre-pandemic period of 17.3 h (*P* < 0.001). Furthermore, the incidence rates of periappendiceal abscess, appendiceal perforation, and gangrene were all notably higher than those recorded before the pandemic (20.7% vs. 4.8%, *P* < 0.001; 17.2% vs. 4.8%, *P* < 0.003; 13.8% vs. 3.8%, *P* < 0.05, respectively) (20).

Appendiceal wall perforation may progress to abscess formation or severe peritonitis, resulting in localized and generalized peritoneal inflammation and abdominal muscle tension, which can lead to severe diffuse abdominal pain. If not treated in a timely manner, the condition may progress to bacterial translocation from the intestines into the bloodstream, leading to septic shock, circulatory failure, and ultimately death (13, 21). We believe that UCAA and CAA represent different stages of the same disease process. Therefore, during the early phase of appendicitis, i.e., before the development of gangrene or perforation, treatment with antibiotics alone can be effective. However, once the condition progresses to gangrenous appendicitis or perforation occurs, surgical intervention becomes necessary to completely remove the source of infection and control the inflammatory response (1, 2, 22).

A body temperature ≥ 37.45 °C, neutrophil percentage ≥ 82.7%, and CRP level ≥ 4.3 mg/L could also serve as important laboratory parameters for predicting CAA. This study revealed that patients with CAA, characterized by gangrenous or perforated forms, exhibited significantly higher body temperatures compared to those with UCAA, a finding that aligns with previous research. In clinical practice, close monitoring of body temperature changes is essential among elderly patients diagnosed with CAA. A marked elevation in body temperature typically signifies a worsening of the infection, necessitating prompt symptomatic intervention (23, 24). Peripheral blood biomarkers also hold considerable clinical significance in the diagnosis and pathological classification of AA. An elevated neutrophil count is associated with the severity of appendicitis; however, numerous inflammatory conditions can lead to increased neutrophil levels, making this marker relatively non-specific. Consequently, additional diagnostic factors must be taken into account for the accurate identification of AA (22, 25). CRP plays a significant role in opsonization, enhancing the activity and motility of phagocytes and thereby promoting the phagocytosis of various pathogens and foreign particles. During episodes of tissue injury, infection, or inflammation, CRP synthesis is upregulated in hepatocytes. The elevation of CRP levels is positively correlated with the severity of the inflammatory or infectious process. As a non-disease-specific acute-phase inflammatory marker, CRP is not directly influenced by commonly used anti-inflammatory or immunosuppressive drugs. Furthermore, its levels remain unaffected by various clinical factors such as fever, elevated erythrocyte sedimentation rate, and increased WBC count. In the diagnosis and disease severity assessment of AA, CRP demonstrates greater clinical utility compared to serum WBC count and neutrophil levels (26, 27).

An analysis of data from the patients with CAA indicated that the presence of nausea/vomiting, WBC ≥ 14.24 × 10^9^/L, neutrophil percentage ≥ 84.3%, and open appendectomy were independent risk factors for postoperative complications. Preoperative nausea and vomiting are common clinical manifestations among patients with appendicitis, potentially attributed to pathophysiological mechanisms such as systemic inflammatory response and gastrointestinal motility disturbances. These symptoms may exert a multifactorial influence on the occurrence and severity of postoperative complications. Preoperative vomiting may result in the aspiration of gastric contents, which can lead to aspiration pneumonia. Frequent vomiting may result in hypovolemia, hypokalemia, and other fluid and electrolyte imbalances, which can impair postoperative recovery. Additionally, persistent vomiting may indicate a more serious underlying abdominal infection and can indirectly contribute to an increased risk of postoperative infections (28, 29). The WBC and neutrophil count in peripheral blood are key laboratory indicators that reflect the systemic inflammatory response and the severity of appendicitis. These parameters are also significantly associated with the risk of postoperative infection complications (30, 31). For elderly patients with CAA who present with the aforementioned risk factors, enteral or parenteral nutrition should be proactively administered during the perioperative period to address dehydration and electrolyte imbalances, and appropriate antibiotics should be promptly administered to control infections.

With the advancement of laparoscopic techniques and the accumulation of surgical experience, laparoscopic surgery has been increasingly applied in the treatment of CAA, yielding significant therapeutic outcomes (1, 32). It is gradually evolving into a more systematic and mature approach. Compared to open appendectomy, laparoscopic surgery offers notable advantages, including reduced trauma, decreased intraoperative blood loss, accelerated postoperative recovery, and a lower incidence of complications. Especially when accessing the appendix in rare and clinically challenging locations, traditional open appendectomy inflicts considerable trauma on the patients, resulting in an extended postoperative recovery period. The associated complications, such as surgical site infections and intestinal obstruction, often contribute to less favorable prognoses for elderly patients with CAA. Therefore, laparoscopic surgery demonstrates superior therapeutic outcomes in the treatment of CAA compared to open appendectomy, making it a favorable option for clinical application and worthy of broader adoption (32, 33).

This study is subject to certain limitations. The single-center origin of the case data and the retrospective study design may compromise the level of evidence and affect the reliability of the analysis results. In addition, the time span for case inclusion is relatively extensive, potentially introducing a degree of bias. Although all the surgeries in this study were performed by experienced physicians, variations in surgical techniques and procedural approaches across different teams may exert a certain impact on the surgical outcomes. In order to further validate and expand upon the findings of this study, more high-quality, multicenter, and large-sample prospective studies should be conducted in the future.

## 5 Conclusions

This study identified the risk factors associated with the development of CAA and postoperative complications among elderly patients (>60 years) by conducting a retrospective analysis of large-scale clinical data. Abdominal signs and symptoms, the presence of appendicoliths, body temperature, and the levels of neutrophils and CRP were associated with the occurrence of CAA, while the surgical approach and the levels of WBCs and neutrophils were associated with postoperative complications. The findings of this study will facilitate the early identification of elderly patients with AA who are suitable candidates for surgical intervention, thereby offering valuable insights into the clinical management and treatment strategies for AA in this specific population.

## Data Availability

The raw data supporting the conclusions of this article will be made available by the authors, without undue reservation.
